# Optimizing genomic selection of agricultural traits using K-wheat core collection

**DOI:** 10.3389/fpls.2023.1112297

**Published:** 2023-06-14

**Authors:** Yuna Kang, Changhyun Choi, Jae Yoon Kim, Kyeong Do Min, Changsoo Kim

**Affiliations:** ^1^ Department of Crop Science, Chungnam National University, Daejeon, Republic of Korea; ^2^ Wheat Research Team, National Institution Crop Sciences, Wanju-gun, Republic of Korea; ^3^ Department of Plant Resources, Kongju National University, Yesan, Republic of Korea; ^4^ Department of Smart Agriculture Systems, Chungnam National University, Daejeon, Republic of Korea

**Keywords:** *Triticum aestivum*, genome-wide association study, genomic breeding, n-fold validation, quantitative trait, qualitative trait

## Abstract

The agricultural traits that constitute basic plant breeding information are usually quantitative or complex in nature. This quantitative and complex combination of traits complicates the process of selection in breeding. This study examined the potential of genome-wide association studies (GWAS) and genomewide selection (GS) for breeding ten agricultural traits by using genome-wide SNPs. As a first step, a trait-associated candidate marker was identified by GWAS using a genetically diverse 567 Korean (K)-wheat core collection. The accessions were genotyped using an Axiom^®^ 35K wheat DNA chip, and ten agricultural traits were determined (awn color, awn length, culm color, culm length, ear color, ear length, days to heading, days to maturity, leaf length, and leaf width). It is essential to sustain global wheat production by utilizing accessions in wheat breeding. Among the traits associated with awn color and ear color that showed a high positive correlation, a SNP located on chr1B was significantly associated with both traits. Next, GS evaluated the prediction accuracy using six predictive models (G-BLUP, LASSO, BayseA, reproducing kernel Hilbert space, support vector machine (SVM), and random forest) and various training populations (TPs). With the exception of the SVM, all statistical models demonstrated a prediction accuracy of 0.4 or better. For the optimization of the TP, the number of TPs was randomly selected (10%, 30%, 50% and 70%) or divided into three subgroups (CC-sub 1, CC-sub 2 and CC-sub 3) based on the subpopulation structure. Based on subgroup-based TPs, better prediction accuracy was found for awn color, culm color, culm length, ear color, ear length, and leaf width. A variety of Korean wheat cultivars were used for validation to evaluate the prediction ability of populations. Seven out of ten cultivars showed phenotype-consistent results based on genomics-evaluated breeding values (GEBVs) calculated by the reproducing kernel Hilbert space (RKHS) predictive model. Our research provides a basis for improving complex traits in wheat breeding programs through genomics assisted breeding. The results of our research can be used as a basis for improving wheat breeding programs by using genomics-assisted breeding.

## 1 Introduction

Common wheat (*Triticum aestivum L.*) is a major staple food crop widely cultivated in many parts of the world. Genetic improvements are urgently required in wheat in order to achieve better quality, higher yields, better adaptation to diverse environments, tolerance to biotic stresses and to meet the needs of a growing population as well as the effects of global climate change (Atlin et al., 2017). It is essential to sustain global wheat production by utilizing accessions in wheat breeding. It is, therefore, fundamental to sustaining global wheat production. The establishment of a core collection or a mini core collection (mini CC) representing the entire genetic diversity of wheat and its relatives in order to find accessions with desirable traits to engineer new varieties, is useful for breeding purposes (Frankel, 1984; van Hintum et al., 2000; Worland, 2001; Zhang et al., 2011; Kumar et al., 2020). In particular, there is a tremendous lack of genome-wide genotypic information due to the wheat genome’s characteristics. There is a large genome in wheat with a size of approximately 16 Gb and has been assembled into 14.5 Gb (Arumuganathan and Earle, 1991; IWGSC et al., 2018). It is an allohexaploid with three homoeologous genomes (2n = 6x = 42, genome formula AABBDD) originating from three ancestral parental species(Sorrells et al., 2003; Gill et al., 2004). The large size and polyploidy-related complexity of wheat collections made genomic analyses difficult to detect the genome-wide molecular diversity of each accession and to determine the population structure of wheat collections (IWGSC et al., 2018).

In spite of this, advances in next-generation sequencing technology are providing a variety of resources for wheat breeding, including high-quality genomic data (IWGSC et al., 2018). A number of high-throughput single nucleotide polymorphisms (SNPs) arrays have been developed and utilized in wheat, including 9K (Cavanagh et al., 2013), 50K (Rasheed and Xia, 2019), 820K (Winfield et al., 2016), 660K (Sun et al., 2020), and 35K (Allen et al., 2017). Detailed information about different arrays has been discussed in previous papers(Bassi et al., 2016; Sun et al., 2020). These SNP arrays were used for genomic-wide association studies (GWAS) and genomic selection (GS) in the United States elite wheat breeding genotype, the International Maize and Wheat Improvement Center (CIMMYT) spring wheat breeding program, and European winter, and spring wheat(Wang et al., 2014; Jin et al., 2016; Winfield et al., 2016; Cui et al., 2017; Rasheed and Xia, 2019; Yang et al., 2019). Marker-associated selection (MAS) is conducted using the SNP data obtained through genotyping-by-sequencing and SNP arrays(Hayashi et al., 2004; Uauy, 2017; Li et al., 2018). It is possible through GWAS to identify individuals associated with a target trait by finding specific markers associated with that trait (Visscher et al., 2017; Uffelmann et al., 2021). GWAS was successfully used for quantitative trait loci (QTL) mapping of wheat properties, such as stress resilience, disease resistance, flowering time and grain yield, using various molecular marker systems (Crossa et al., 2007; Bentley et al., 2014; Bhatta et al., 2018). Although many agricultural traits have been studied extensively and their markers identified, other traits, such as the awn traits that could be used to improve wheat grain yields, have rarely been studied (Goddard, 2009; Sukumaran et al., 2018; Sheoran et al., 2019; Krishnappa et al., 2021).

GS is a new MAS form that offers efficiency gains over phenotypic selection or conventional MAS. A MAS is an indirect selection process in which individuals are selected according to a trait of interest(Fernando and Grossman, 1989). However, MAS is only practical when a given trait is governed by a single gene or a small number of genes, whereas such an approach would be difficult or irrelevant for quantitative traits (i.e., traits governed by tens or hundreds of minor genes)(Bernardo, 2008). In terms of short and long-term responses, GS was reported to obtain more considerable gains from selection than MAS based on only a few significant markers. As part of the GS process, a training population (TP) of relevant individuals is developed, which is a population that consists of individual genotypes and phenotypes. Based on this information, it is possible to develop a model that uses phenotypes as responses and genotypes as predictors, based on the effects of dense markers distributed across the genome on the net genetic merit of an individual. An estimated individual effect of each marker is estimated, and the additive sum of all the markers effects is used to calculate each individual’s genomic-estimated breeding value (GEBV)(Meuwissen et al., 2001). Further, genomic selection has the potential to increase gain per unit of cost due to recent advances in genotyping that enable thousands of marker data points to be generated more economically and rapidly than was previously possible (Lorenz et al., 2012). At the same time, phenotyping remains time and labor-intensive. GS was extensively studied in animal breeding to accelerate the rate of gain for quantitative traits, and it is becoming more widely adopted in plant breeding. The accuracy of GS prediction is determined by the correlation between GEBV and trait phenotype(Wolc et al., 2011). In wheat, GS was assessed for breeding important quantitative traits such as grain yield, quality traits such as flour yield, flour protein, solvent retention capacity for sucrose, lactic acid, water absorption, sodium carbonate, and softness equivalent, as well as resistance to Fusarium head blight (FHB) and stem rust (Heffner et al., 2011a; Heffner et al., 2011b; Rutkoski et al., 2015; Arruda et al., 2016; Guzman et al., 2016).

The purpose of this study is to conduct two genomics-assisted breeding approaches (GWAS, GS) with the K-wheat core collection (CC) with genetic diversity for ten agricultural traits. In the first step, a mini core collection (miniCC) was constructed based on SNP markers across the Kwheat genome. We then used the miniCC to identify trait-associated markers using the fixed and random model circulating probability unification (FarmCPU). The third step involved evaluating the prediction accuracy with the CC using various compartmentalized training sets and statistical models. This study will serve as a foundation for the development of improved wheat varieties that are more efficient than conventional breeding methods.

## 2 Materials and methods

### 2.1 Plant materials

**Figure 1:**
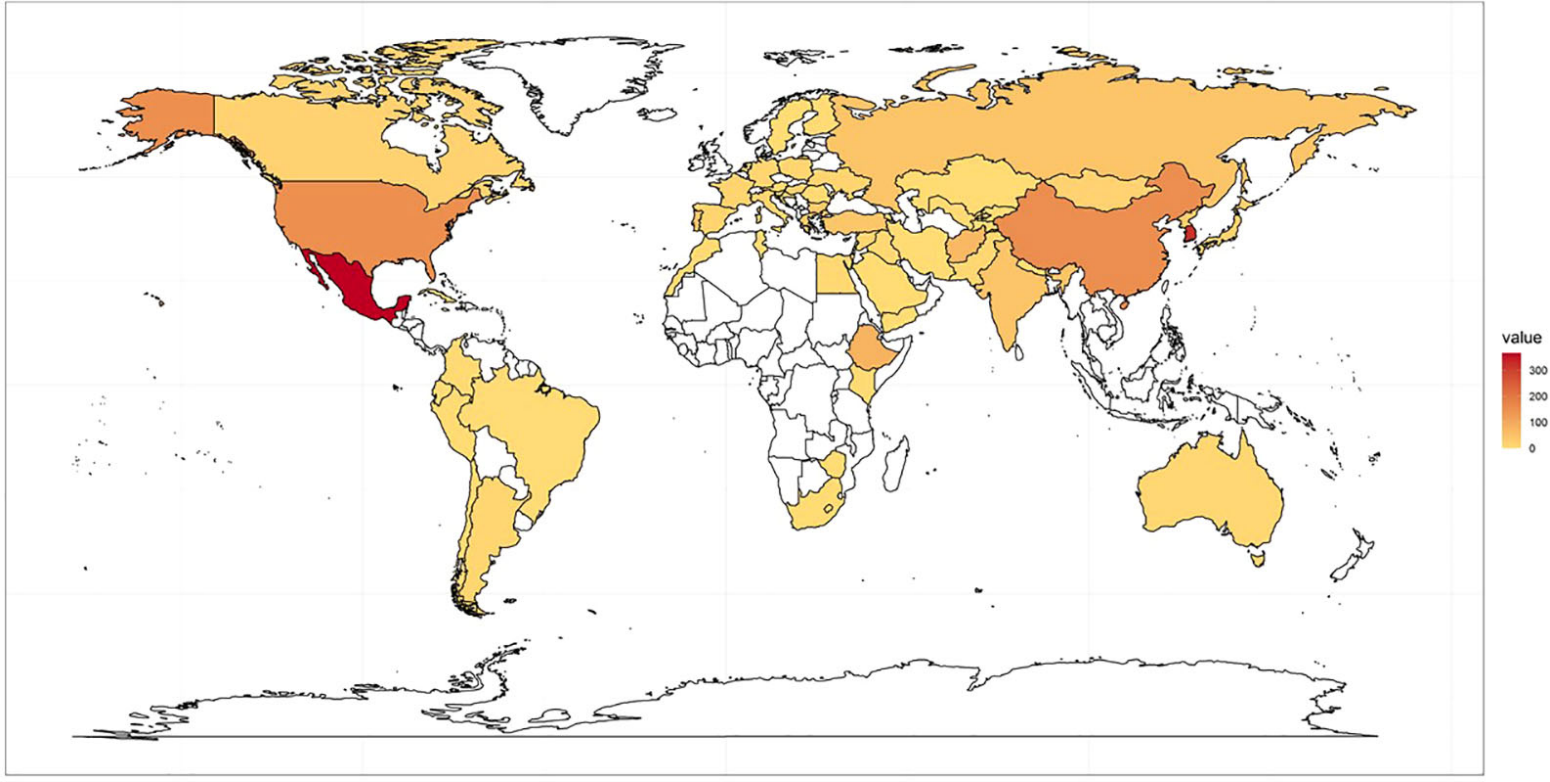
567 accessions of K-wheat core collection used in this study and country of origin.

The K-wheat core collection (CC) reported in previous studies was used as a training population for this study (Min et al., 2021). The CC consists of accessions collected worldwide and stored in National Agrobiodiversity Center (http://genebank.rda.go.kr/). Based on 37 simple-sequence repeat (SSR) markers, this CC includes 567 accessions from 49 countries. (**Figure 1**; **Supplementary 1**).

### 2.2 Genotyping and built of K-wheat mini-core collection

Since the CC was established based only on 37 SSRs (Balfourier et al., 2007), it cannot scan the entire genome of wheat. Therefore, the CC genotype was further determined with the Axiom^®^ 35k breeders SNP array (Affymetrix, CA, USA) (Allen et al., 2017) to screen the entire genome and re-analyze subpopulation structure. Genotyping was performed on an Affymetrix GeneTitan system following Affymetrix’s manual (Axiom^®^ 2.0 Assay Manual Workflow User Guide Rev3). SNP callings were performed using modified versions of Affymetrix Power Tools (APT) and SNPolisher™(Affymetrix, CA, USA) to account for the wheat genome’s specificity. Genotype scoring was performed in Affymetrix Genotyping Console using recommended QC metrics (0.82 DQC, 97 QC call rate) (Przewieslik-Allen et al., 2021). Among 35,143 SNPs, SNPs with a minor allele frequency (MAF) of 5% or less were removed.

Genocore (Jeong et al., 2017) and CoreHunter (Thachuk et al., 2009) programs were used to select the K-wheat mini CC based on the whole genome SNPs. Genocore selected subsets using -d 0.001 and -cv 200 parameters. Core Hunter selected subsets using default options.

### 2.3 Population structure analysis

STRUCTURE 3.4.0 software (Pritchard et al., 2010) was used to analyze population structure. There were 50,000 burn-in iterations, followed by 100,000 Markov Chain Monte Carlo (MCMC) iterations after a burn-in of each run. The hypothetical number of subpopulations (k) was preset from 2 to 9. STRUCTURE HARVESTER (Earl, 2012) was used to identify the best k. SNPs were considered for phylogenetic analysis using the SNPhylo pipeline to generate phylogenetic trees by the maximum likelihood method (Lee et al., 2014). Multivariate analysis was performed using the principal component analysis (PCA) of the three components of Tassel v.5.2.5 (Bradbury et al., 2007). The PCA was constructed based on individual eigenvectors. PCA plots were classified according to subgroups of the population structure analysis.

### 2.4 Phenotype and statistical analysis

Agricultural traits of 567 accessions were measured at the National Institute of Crop Science research field (35° 49’ 48.235”N, 127° 2’ 27.183”E). Ten agricultural traits were measured from 2018 to 2019, and the accumulated data was quantified for each trait. Agricultural traits were: awn color (AC), awn length (AL), culm color (CCL), culm length (CL), ear color (EC), ear length (EL), leaf length (LL), leaf width (LW), days to heading (HD), and days to maturity (MD).

The bar plot depicts the phenotypic data distribution for all ten traits. Correlation analyses between phenotypes were performed using Pearson correlation coefficients of mini-tab 16.2.4 software (Minitab, 2021). The phenotype data used for association analysis was calculated by best linear unbiased prediction (BLUP) using the phenotype package of the R program (Piepho et al., 2008).

### 2.5 Association analysis

Association analysis was performed using the FarmCPU method in the Genome Association and Prediction Integrated Tool (GAPIT) R package (Lipka et al., 2012). A false discovery rate (FDR) threshold adjusted -log10 *P* > 3 was used to state significant marker-trait associations. Significant SNPs were annotated using Variant Effect Predictor of Ensembl-plants.

In order to find each trait-associated candidate gene, a gene region of up- and down-stream of 500 kb flanking sequences was secured in a significant SNP. For the flanking sequences of significant SNPs, the Basic Local Alignment Search Tool (BLASTx) analysis was performed using the National Center for Biotechnology Information’s (NCBI) nr protein database (confined to Viridiplantae) as a subject (https://www.ncbi.nlm.nih.gov/genbank/).

### 2.6 Prediction model for genomic selection

The prediction ability of Bayes A, ridge regression (equivalent to G-BLUP), least absolute shrinkage and selection operator (LASSO), random forest regression (RF), support vector machine (SVM), and reproducing kernel Hilbert space (RKHS) models widely used in various crops were evaluated and comparatively analyzed. All models are embedded in the R package Breed Wheat Genomic Selection Pipeline (BWGS) (Charmet et al., 2020). Bayes A uses a scaled-t prior distribution of marker effects (Neal, 2012), and genomic best linear unbiased prediction (GBLUP) uses a marker-based relationship matrix (Endelman, 2011). LASSO is a penalized regression method. RF uses a regression model on tree nodes based on bootstrapping data and assumes that interactions between markers can be captured (Breiman, 2001). RKHS is based on genetic distance and kernel function to control the distribution of marker effects and is effective in detecting non-additive effects (Pérez and de Los Campos, 2014). For all those models, ten-fold cross-validation was used to test the credibility of GEBV values.

In order to confirm the prediction ability according to the TP, the accessions included in the CC were divided into randomly selected subgroups or subgroups based on genetic backgrounds. First, the randomly selected subgroups were repeated ten times by selecting as many individuals as 10% (56 accessions), 30% (170 accessions), 50% (284 accessions), 70% (397 accessions), and 100% (567 accessions) of the CC. In order to see the differences according to the genetic backgrounds, three subgroups divided based on the results of the population structure analysis were used as training populations.

### 2.7 Validation of prediction models

Thirty-five Korean wheat cultivars were used to verify the estimated prediction accuracy. The genotype data of the breeding population was obtained with an Axiom^®^ 35k breeders SNP array. The phenotype data were measured by the NICS (35° 49’ 48.235”N, 127° 2’ 27.183”E) from 2018 to 2019. The prediction ability was verified through correlation analysis between the GEBV and the measured values of the breeding population. The bwgs.predict, the function of the R package BWGS, was used for verification.

## 3 Results

### 3.1 Construction of a K-wheat mini-core collection

**Figure 2:**
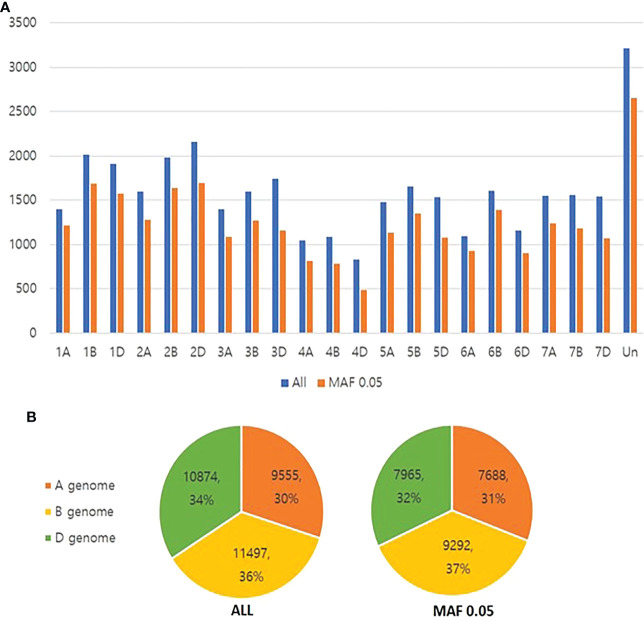
**(A)** SNP distribution by chromosome. **(B)** SNP distribution by subgenomes.

SNP genotyping for 576 accessions included in the CC constructed using SSR markers was performed using the Axiom^®^ 35k breeders SNP array (Affymetrix, CA, USA) to construct the K-wheat mini CC and to re-analyze subpopulation structures. Since the original CC was constructed based on 37 SSR markers, the ability to scan the entire genome would be weak. Instead, the mini CC may enhance the power of predictive breeding because the 35k SNP array may efficiently cover the whole genome of bread wheat. Since SNP calling was not made in nine out of 576 accessions, the SNP data of 567 accessions was used for downstream analyses. A total of 35,153 SNPs were obtained through a 35K wheat DNA array. Across all the wheat chromosomes, an average of approximately 1,597 SNPs were identified in each chromosome. In terms of SNP distribution by chromosome, the 4D chromosome had the smallest number (828), and the 2D chromosome had the largest number (2,156). When filtering based on the MAF of 0.05, 27,598 SNPs were determined. Even after filtering, the 4D chromosome had the smallest number of SNPs, and the 2D chromosome had the largest (**Figure 2**). 4D chromosome had smallest number because it has the lowest number of genes and probes.

Two software packages, Core Hunter and Genocore, were used to establish the mini CC, resulting in 113 and 216 accessions, respectively. Among them, 82 accessions were selected by both programs. Therefore, the mini CC consisting of 247 accessions was finally determined (**Supplementary Table 1**).

### 3.2 Genetic diversity and population structure

Population structure and phylogeny tree analysis were performed to investigate the genetic diversity of the CC with SNPs. The number of subgroups in the CC was determined by model-based structure analysis with model parameters k from 2 to 9 (**Supplementary Figure 1**). The maximum likelihood values of the CC showed a typical curvilinear response to increasing k, such that k = 3 was defined to provide the optimal structure for further analysis (**Supplementary Figure 1A**). CC-sub 1 comprised 125 accessions with the genetic diversity index of 0.405 based on the fixation index (Fst). CC-sub 2 comprised 269 accessions with the genetic diversity index of 0.397, and CC-sub 3 comprised 173 accessions with the genetic diversity index of 0.41. A phylogeny tree was built using the common SNPs markers of CC to better detail the kinship among the accessions. The phylogeny tree of the CC showed two main clusters with a robust separation between them (**Supplementary Figure 1B**). Within each cluster, accessions were mainly grouped in agreement with the groups obtained previously by the population structure analysis. However, the three subgroups were mixed without forming a cluster when the PCA analysis results were divided by the population structure (**Supplementary Figure 1C**).

**Figure 3:**
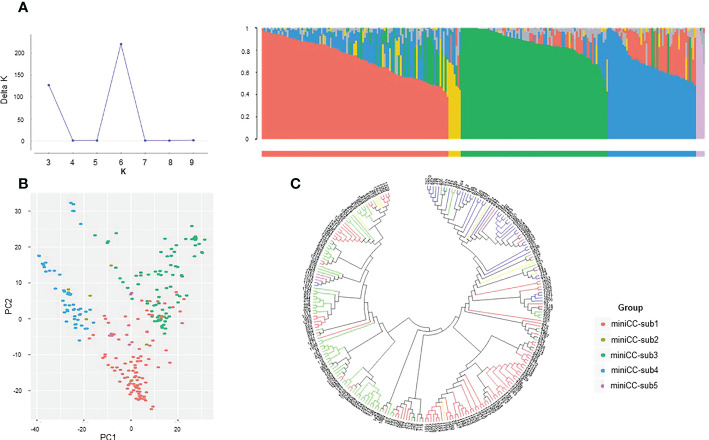
Population structure analysis of K-wheat mini-core collection using SNP markers. **(A)** Results of Evanno test and population structure of the mini-core collection. **(B)** Principal component analysis (PCA) of a mini-core collection based on SNP markers. Based on the subpopulation structures, the red, yellow, green, blue, and purple dots represent mini-sub 1, mini-sub 2, mini-sub 3, mini-sub 4, and mini-sub 5, respectively. **(C)** Molecular phylogenetic analysis by Maximum Likelihood method using the mini-core collection. Phylogenetic analysis was inferred by the Maximum Likelihood method based on the Tamura-Nei model. Phylogeny tree analyses were conducted by MEGA7 (Kumar et al., 2016). Based on the subpopulation structures, the red, yellow, green, blue, and purple lines indicate mini-sub 1, 2, 3, 4, and 5, respectively.

For the mini CC, Evanno test showed 6 clusters with K=6. (**Figure 3A**). However, there was no significant difference in the value in one cluster among the six clusters. Therefore, they were not differentiated into distinct groups and were divided into five groups. Mini CC-sub 1 comprised 104 accessions with the genetic diversity of 0.411 based on the fixation index (Fst). Mini CC-sub 2 consisted of seven accessions with the genetic diversity index of 0.389, and mini CC-sub 3 comprised 82 accessions with the genetic diversity index of 0.371. Mini CC-sub 4 consisted of 49 accessions with the genetic diversity index of 0.399, and mini CC-sub 5 consisted of five accessions with the genetic diversity index of 0.383. The phylogeny tree of the mini CC showed three main clusters with a robust separation from each other (**Figure 3B**). Within each cluster, accessions were mainly grouped in agreement with the groups obtained previously by the population structure analysis. The PCA analysis clearly distinguished three subgroups (mini-subs 1, 3, and 4) (**Figure 3C**).

### 3.3 Correlation analysis of phenotype

**Figure 4:**
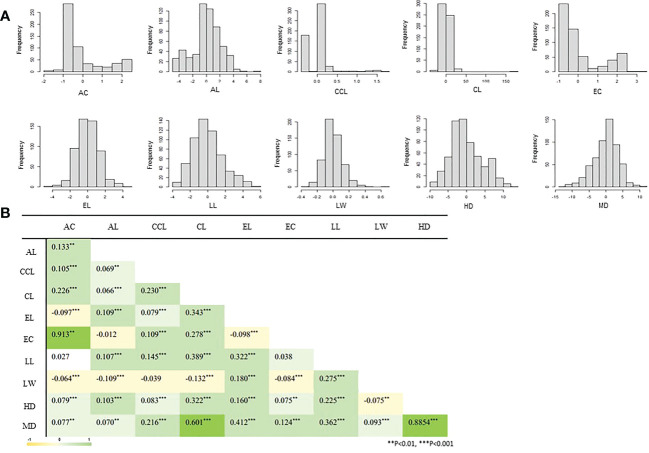
Correlation analysis and frequency distribution of the phenotypes in the K-wheat core collection. AC, awn color; AL, awn length; CCL, culm color; CL, culm length; EC, ear color; EL, ear length; LL, leaf length; LW, leaf width; HD, days to heading; MD, days to maturity. **(A)** It means the distribution of phenotypes of all accessions. **(B)** Shows the correlation of each phenotype. All correlation analyzes are expressed as Pearson's correlation coefficient.

The frequency distribution of ten agricultural phenotypic data of 567 accessions included in the CCL is shown in **Figure 4A**. As a result of correlation analysis between each trait by Pearson correlation coefficient analysis (**Figure 4B**), a significant and strong positive correlation (0.913) was found between EC and AC. There was also a significant strong positive correlation (0.8854) between HD and MD. In addition, weak positive correlations between many traits were observed. For the LW example, a weak negative correlation was observed with the AC, AL, CCL, CL, and EC traits (-0.1 ~ -0.039).

### 3.4 Marker-trait association analysis

**Figure 5:**
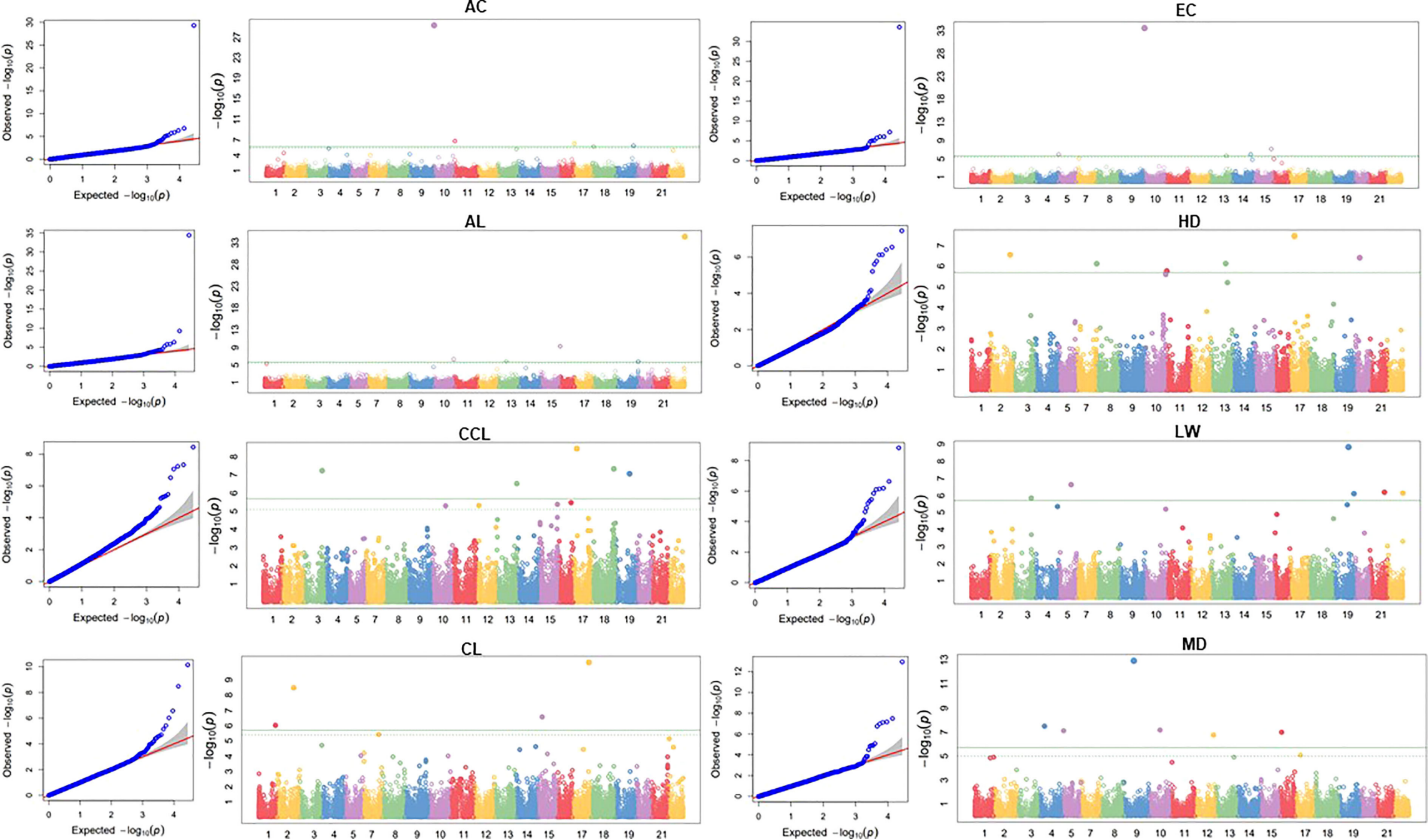
Q-Q plots and Manhattan plots of genome-wide association study (GWAS) for ten agronomic traits of the K-wheat mini-core collection. The x-axis represents the wheat chromosomes, and the y-axis indicates statistical significance according to -log_10_ (p-value). AC, awn color; AL, awn length; CCL, culm color; CL, culm length; EC, ear color; LW, leaf width; HD, days to heading; MD, days to maturity.

Marker-trait association (MTA) of ten traits was conducted using FarmCPU models (**Figure 5**). The SNPs with FDR adjusted -log10(P) > 3 were designated as significant. Significant SNPs detected for each trait using the FarmCPU model are detailed in **Table 1**. A total of 18 significant SNPs were identified for eight traits. For the AC trait, one significantly associated SNPs were distributed in chr1B. For the AL trait, two significant SNPs were identified in chr4D and chr2D, and four significant SNPs were identified in four chromosomes for the CCL trait. Two significant SNPs were identified for the CL trait in chr1B and chr2D. For the EC trait, two significant SNPs were detected in chr1B and chr2D. For the HD trait, one significant SNP was detected in chr3D, and six SNPs were detected in six chromosomes for the MD trait. SNP AX-94454667, located in chr1B, was identified in both AC and EC traits. The differences in the phenotypes of individuals according to the alleles of the identified significant SNP markers were confirmed. In the case of AC, the average phenotypic value of accessions with the A allele was 2.27, ranging from yellow to yellow-brown, whereas the average phenotypic value of accessions with the C allele was 4.47, ranging from brown to reddish-brown. In the MTA results of AL, the mean phenotypic value of accessions with the T allele of SNP AX-94613491 was 5.9 cm, while that of accessions with the C allele was 3.1 cm. Based on the relationships between the phenotypic values and alleles listed above, one can select a wheat plant with about 2.8 cm of awn length. It could have an A allele in the designated locus. Likewise, the mean phenotypic value of accessions with the A allele of SNP AX-94937575 was 5.43 cm, while that of accessions with the G allele was 4.9 cm. Accessions with the T allele of SNP AX-94613491 and the A allele of AX-94937575 had an average length of 6.41 cm. Accessions with the T allele of SNP AX-94613491 and the G allele of AX-94937575 had an average length of 5.7 cm. Accessions with the C allele of SNP AX-94613491 and the A allele of AX-94937575 had an average length of 3.02 cm. Accessions with the C allele of SNP AX-94613491 and the G allele of AX-94937575 had an average length of 3.55 cm. Therefore, the allele of SNP AX-94613491 is more likely to be related to length than SNP AX-94937575. In the MTA results of CL, the mean length of accessions with the A allele of SNP AX-94638909 was 90 cm, whereas the mean length of accessions with the G allele was 61 cm. In the MTA results of ear color, the average phenotypic value of accessions with the A allele was 1.79, ranging from yellow-white to yellow. In contrast, the average phenotypic value of accessions with the C allele was 4.5, ranging from brown to reddish-brown. In the MTA results of days to heading, accessions with the A allele of SNP AX-94881841 had an average of 19 days, and those with the G allele had an average of 21. The information can be utilized for developing selection markers for wheat breeding programs.

**Table 1:** Significant SNPs identified for 8 agricultural traits of mini-core collection.

SNP	Chr	allele^a^	Position^b^	Maf^c^	effect^d^	*P*-values^e^	Consequence	Gene ID^f^
Awn color								
AX-94454667	chr1B	A/C	1942165	1.5E-01	0.98	24.85	Synonymous	TraesCS1B02G002900
Awn length								
AX-94613491	chr4D	T/C	509666570	3.4E-01	1.76	29.94	Synonymous	TraesCS4D02G365800
							Upstream	TraesCS4D02G365800
AX-94937575	chr2D	A/G	648547627	3.0E-01	0.84	5.13	Intergenic	
Culm color								
AX-94445918	chr3D	A/T	113799234	5.0E-01	0.98	4.00	3’ UTR	TraesCS2B02G541300
AX-94428945	chr2B	T/G	737635801	3.5E-01	0.14	3.26	Intron	TraesCS3D02G148600
AX-95005487	chr4A	A/G	614244697	7.1E-02	-0.27	3.26	Upstream	TraesCS4A02G328900
							3’ UTR	TraesCS4A02G329000
AX-94921816	chr5A	A/G	462808499	6.7E-02	-0.26	3.22	3’ UTR	TraesCS5A02G248500
Culm length								
AX-94816988	chr3D	T/G	547625708	3.6E-01	24.49	5.70	Downstream	TraesCS3D02G435300
							3’ UTR	TraesCS3D02G435400
AX-94638909	chr7A	A/G	542708859	9.9E-02	-6.46	4.34	Synonymous	TraesCS7A02G369100
Ear color								
AX-94454667	chr1B	A/C	1942165	1.5E-01	1.35	29.11	Synonymous	TraesCS1B02G002900
AX-94508473	chr2D	A/G	567967948	1.8E-01	0.54	3.05	Intergenic	
Days to heading								
AX-94881841	chr3D	A/G	136402016	3.6E-01	3.06	3.01	Synonymous	TraesCS3D02G164700
							Downstream	TraesCS3D02G164800
							Downstream	TraesCS3D02G164800
Leaf width								
AX-94756234	chr5A	A/G	481901482	4.9E-01	-0.05	4.39	Upstream	TraesCS5A02G271400
							Intron	TraesCS5A02G271500
							Missense	TraesCS5A02G271500
Days to maturity								
AX-94509831	chr3B	A/C	317628294	1.9E-01	-2.29	8.50	Upstream	TraesCS3B02G226800
AX-94954171	chr3A	A/G	159839248	1.2E-01	1.82	3.35	Intron	TraesCS3A02G159900
AX-94841333	chr1B	T/C	334681540	1.6E-01	-1.42	3.27	Missense	TraesCS1B02G187300
AX-95186852	chr1A	T/C	27362485	1.7E-01	1.28	3.27	Missense	TraesCS1A02G046100
							Missense	TraesCS1A02G046100
AX-94442558	chr1D	A/C	43124061	4.8E-01	-0.92	3.24	Synonymous	TraesCS1D02G063000
AX-95222044	chr4B	A/G	598261950	2.8E-01	-0.84	3.09	Stop gained	TraesCS4B02G307900

a) major allele/minor allele.

b) Position in IWGSC RefSeq v1.0.

c) Minor allele frequency.

d) the estimated effect of replacing minor allele by major allele.

e) FDR-corrected p values after Bonferroni correlation.

f) Genes annotated with high confidence by IWGSC.

### 3.5 Identification of putative candidate genes

Candidate genes were identified for the significant SNPs associated with eight traits (**Table 1**). SNP AX-94454667 was associated with the AC trait and the EC trait was marked with cytochrome P450. One significant SNP of unknown function was additionally identified in the ear color trait. In the significant SNP of the culm color trait, the potassium channel *kat2* gene was annotated, and in the culm length, the *amp* gene and *glb3* gene were annotated. As for the leaf width, the NAC domain-containing protein involved in growth and development was identified. For significant SNPs related to the days to maturity trait, the *pp1* gene and the *tet* gene were annotated. All the genes were somehow related to traits, which will be discussed further in the next section.

### 3.6 Heritability of ten agricultural trait

The narrow-sense heritabilities (H^2^) of ten agricultural traits used for genomic selection are shown in **Table 2**. The heritability of the traits measured in the CCL ranged from 0.004 to 0.8. The highest heritability estimates were for HD (0.8), while the heritabilities of EC and CL were low (less than 0.1).

**Table 2:** Variance components and heritability of 10 agronomic traits in 567 core collection.

Traits	Genetic Variance	Residual variance	Phenotypic variance	Heritability
AC	1.01	2.92	3.92	0.26
AL	1.01	4.67	5.68	0.18
CL	3.27	30.52	33.79	0.10
EC	0.02	4.34	4.36	0.00
EL	1.01	5.91	6.92	0.15
CC	1.01	6.07	7.08	0.14
LL	1.01	6.80	7.82	0.13
LW	1.00	1.66	2.67	0.38
HD	44.39	10.46	54.85	0.81
MD	1.00	1.31	2.31	0.43

AC, awn color; AL, awn length; CC, culm color; CL, culm length; EC, ear color; EL, ear length; LL, leaf length; LW, leaf width; HD, days to heading; MD, days to maturity.

### 3.7 Prediction accuracy comparison

**Figure 6:**
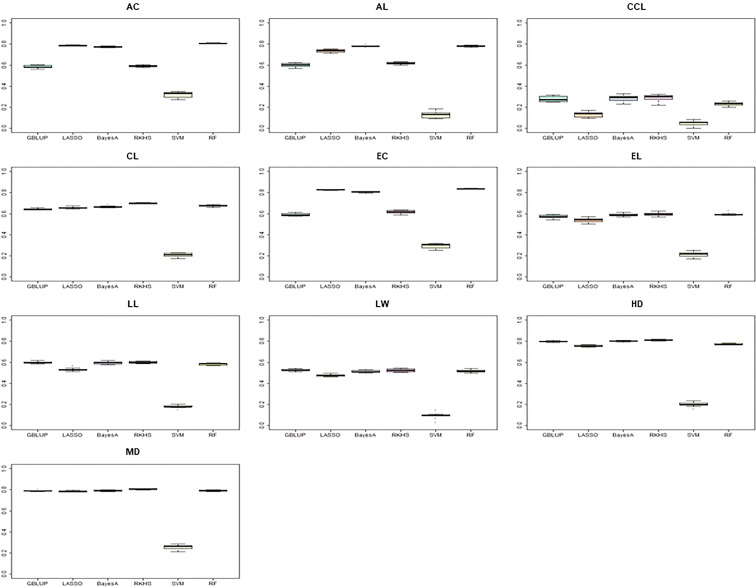
Comparison of prediction accuracy according to different predictive models. The y-axis is the prediction accuracy, and the x-axis is each model. The entire K-wheat core collection was used as a training population. AC, awn color; AL, awn length; CCL, culm color; CL, culm length; EC, ear color; LW, leaf width; HD, days to heading; MD, days to maturity.

To compare the prediction accuracy of each model, we compared six models commonly used in the GS procedure (**Figure 6**). The entire CC was used as an initial TP to calculate the prediction accuracy. The SVM model showed the lowest prediction accuracy for all traits, and RF confirmed the best prediction accuracy for all traits. For the culm color trait, the prediction accuracy of less than 0.4 was determined. The prediction accuracy of 0.4 or more was determined for the other nine traits except for the SVM model.

**Figure 7:**
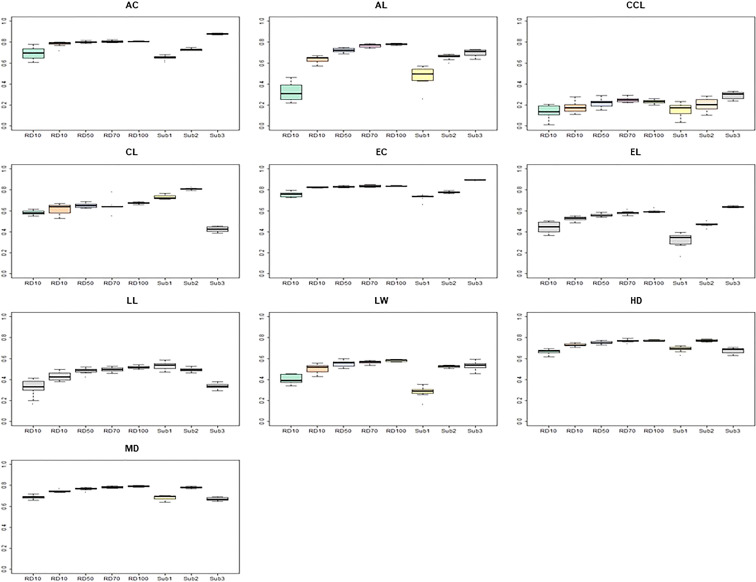
Comparison of prediction accuracy according to compartmentalized training populations. The y-axis is the prediction accuracy, and the x-axis is each training population. The predictive model used for this analyses is RF. AC, awn color; AL, awn length; CCL, culm color; CL, culm length; EC, ear color; LW, leaf width; HD, days to heading; MD, days to maturity.

In order to compare the prediction accuracy according to the composition of the TPs, eight TP sets were constructed (**Figure 7**). It was divided into five TP types (RD10, RD30, RD50, RD70, RD100) by randomly selected accessions based on the proportions of the entire CC and three TP types (CC-sub1, CC-sub2, CC-sub3) according to the subpopulation structure divided by the genetic background. When the TP was randomly selected from all traits, prediction accuracies increased as the size increased. However, in the subgroup based on the population structure, CC-sub2 had the largest number of accessions (269), but it did not always show the best prediction accuracy. The AC, CCL, and EC traits had the highest prediction accuracy in CC-sub 3. In particular, the prediction accuracy of CC-sub 2 was 0.87 for the AC, and the accuracy of CC-sub 3 was 0.89 for the EC. For the AC, EC, HD, and MD traits, the prediction accuracy was above 0.6 using any TP. The AL, LL, and EL traits had similar patterns of prediction accuracy in most TPs.

### 3.8 Validation of breeding populations

**Figure 8:**
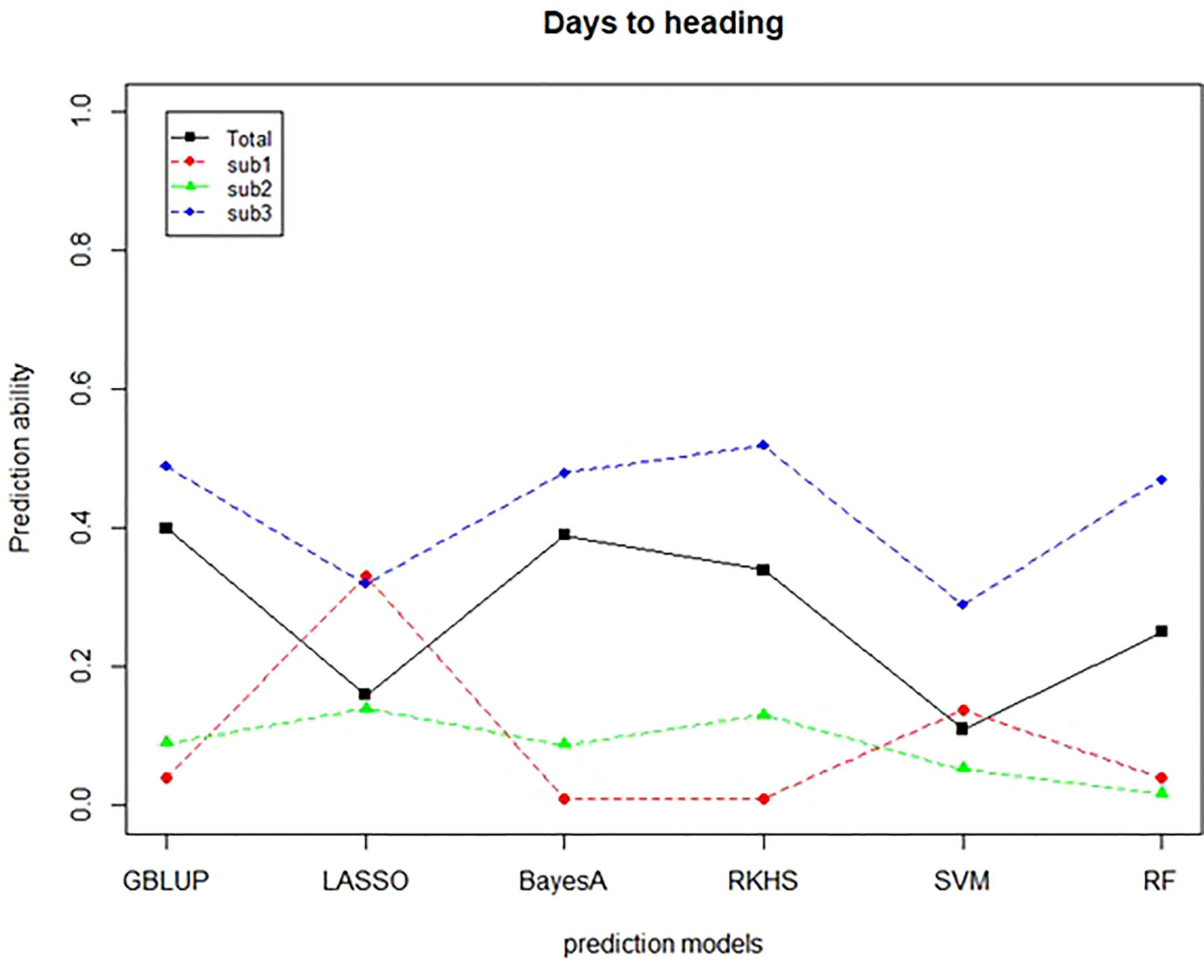
Prediction ability of validation population for days to heading. The predictive ability of six predictive models was verified using four TPs. The X-axis means the prediction model, and the y-axis means the predictive ability confirmed through the comparison between GEBV and BLUP. The black line indicates CC, the red dotted line indicates CC-sub1, the green dotted line indicates CC-sub2, and the blue dotted line indicates CC-sub3.

A further validation was conducted to confirm the predictive ability of the TP of the CC. Thirty-five Korean wheat cultivars with HD data were used as a validation population (VP). The prediction ability was determined by correlating GEBVs with phenotypic data, which is an actual observed value. For the TP for verification, the case where the whole CC was used as a TP and three subgroups based on the subpopulation structure were used. The prediction ability of CC-sub 3 was the highest. A comparison between the models confirmed the prediction ability of 0.49, 0.52, and 0.47 in GBLUP, RKHS, and RF, respectively (**Figure 8**). As a result of using 4 TPs, CC-sub 3 TP had the highest prediction ability. Next, when the CC was used as the TP, the prediction ability of 0.4 was confirmed for the GBLUP and Bayes models.

The GEBV of the RKHS model to CC-sub 3, with the highest prediction accuracy, and BLUP of the actual phenotype were compared (**Table 3**). The 20 cultivars shown in **Table 3** were selected based on GEBVs. Of the ten individuals presumed to have early HD based on the GS procedure, seven individuals were identified as having an early HD per actual phenotypic data. Seven of the ten individuals presumed to have late HD were identified as having a late HD in the actual phenotype. The validation process indicated that the GS was very efficient in selecting desired individuals for breeding with the HD trait

**Table 3:** Comparison of BLUP and GEBV in breeding population.

Cultivar	BLUP	GEBV
**Jokyung**	-0.88	0.69
Hanbaek	-0.42	1.06
Sukang	0.04	1.27
**Joeun**	-2.72	1.59
**Johan**	-1.80	1.63
**KG**	-0.88	2.01
**Jonong**	-2.72	2.07
Yeonbaek	-0.42	2.13
**Jojung**	-1.34	2.13
**Jeokjung**	-1.34	2.13
.	.	.
.	.	.
.	.	.
**Ori**	1.42	3.14
Milsung	0.04	3.15
Hojung	0.96	3.24
**Gobum**	1.42	3.25
**Eunpa**	2.34	3.70
NamH	0.50	4.15
**Topdong**	2.34	4.35
**Grue**	3.26	4.60
**Jinpum**	2.80	4.71
**Cheonggey**	1.42	4.80

Bold text indicates varieties with matching BLUP and GEBV.

## 4 Discussion

Wheat is a major crop, and various varieties are grown in many countries. Breeding requires the development of varieties with characteristics that breeders value as necessary, such as increased grain yield, adaptation to climate change, and disease resistance. The first step in developing these breeds is to secure diverse individuals. Wheat diversity panels, such as core collections, were developed to understand populations’ genetics and structure. A variety of phenotypic variations offered by those panels could be used as breeding resources to develop markers or be directly applied for conventional selective breeding. Recently, various populations with phenotypic variations have been used for predictive breeding with advanced statistical models and computational resources to deal with big genomic data.

According to the Balfourier et al. (Balfourier et al., 2007), a core collection of global bread wheat was built using 38 SSR markers from 3,942 accessions collected from 73 countries. The National Institute of Agrobiological Sciences (NIAS), Japan wheat core collection was created using GBS-based SNPs, but this was limited to varieties in Japan (Takeya et al., 2013). In the current study, 567 core collections generated using 37 SSR markers from the previous studies were reconstructed by genotyping with the Axiom^®^ 35k breeders SNP array (Affymetrix, CA, USA) for the SNPs to cover the entire wheat chromosomes, building the K-wheat mini CC with 247 accessions. A CC corresponds to about 30% of the total, and a mini CC to about 12% of the total. When the CC and the mini CC were divided by geographic origins, it was confirmed that they were composed of a certain percentage (**Supplementary Table 2**). Accessions originating from the African continent accounted for about 5% of the CC and mini CC, and accessions from Asia accounted for about 37–39%. Accessions from Europe accounted for 16–18%, South America for 17–19%, and North America for 8–12%.

To obtain useful information about the genetic diversity and population structure of the CC and mini CC, we divided them into three and six subgroups based on the population structure analysis (**Supplementary Figure 1**; **Figure 3**). The PCA and phylogeny trees of the mini CC were clustered similarly to the subpopulations identified in the population structure analysis. Accessions originating from Korea tended to cluster in Asian countries, including Japan, America, and the majority. Accessions from China tended to be clustered into individual groups. Accessions from Europe tended to be clustered into subgroups. Even in similar regions, there are differences among varieties according to breeding programs for improvement. Therefore, the exchange and utilization of diverse accessions worldwide effectively expanded the genetic basis of wheat breeding (Yang et al., 2020). It is costly and time-consuming to describe agricultural traits or quality characteristics with large-scale wheat accessions due to duplications in terms of their genetic backgrounds. This occurs because some traits or characteristics are present in more than one wheat accession, making the process of describing each one individually produce considerable redundancy in terms of the data collected. In addition, it is difficult to accurately compare the traits of different accessions when their genetic backgrounds are similar. The miniCC was constructed based on SNP markers across Korean wheat. Genetic diversity revealed six subgroups and one admixture group (k=6), which is less than the K-wheat core collection (k=7). The miniCC does not appear to have covered the genetic background of the accessions in the same manner as the K-wheat core collection. As a result, the traits of the accessions in the miniCC may require further investigation in comparison to those of the accessions in the core collection of K-wheat. However, miniCCs with decent genetic diversity can facilitate the identification of trait-related markers or individuals through GWAS since A mini core collection (miniCC) is a smaller, more manageable collection of plant genetic resources that are representative of a larger collection.

We obtained phenotypic data of ten agronomically important traits. First, association analysis using the FarmCPU model was conducted, resulting in 19 SNPs significantly associated with those traits. Significant SNPs associated with traits were selected based on -log 10 P > 3. The Bonferroni correction and FDR correction to avoid false positive or false negative results are very strict. Therefore, it is difficult to select complex traits and quantitative traits based on the existing P-value threshold of 5 × 10^-8^.However, candidate SNPs estimated to be significant in our study require additional validation (Gao et al., 2016). A high positive correlation of 0.913 was shown for the awn color and ear color traits, and in the GWAS results, one significant SNP located on chromosome 1B was shared. SNP AX-94454667, shared by the awn color and ear color traits, was annotated as cytochrome P450, which plays an important role in the biosynthesis of flavonoids and anthocyanins, colored compounds of flavonoids (Tanaka and Brugliera, 2013). Culm length is annotated with the *amp* gene, a disease resistance-related gene that plays an important role in the immune system, and the *glb* gene, which is involved in growth development and stress response by participating in plant oxygen supply. In days to maturity, the *pp1* gene (Máthé et al., 2019) and *tet* gene (Reimann et al., 2017) involved in plant development, environment, and stress signaling pathways were identified. Significant SNP identified in leaf width was annotated with the *NAC* gene family, one of the strongest transcription factor families involved in various processes such as development, aging, and response to environmental stress (Olsen et al., 2005). The significant SNPs associated with days to heading were annotated as the *agc* gene involved in response to environmental stress and immunoregulation (Máthé et al., 2019). In most traits, gene families involved in plant hormones, growth development, and stress response were annotated.

Significant differences in phenotypes according to alleles of SNPs were confirmed. Accessions with the A allele of SNP AX-94454667 had yellow-white to yellow in awn color and ear color. It was confirmed that accessions with the C allele ranged from brown to reddish-brown in both traits. In days to heading, it was confirmed that accessions with the A allele of SNP AX-94881841 were about nine days earlier. The awn length confirmed that the subjects with the T allele of SNP AX-94613491 were 2 cm longer on average. We identified significant SNPs associated with eight previously unknown agronomic traits. However, further studies are needed to validate the markers detected in this study using other populations and environments.

Several researchers have reported genomic selection studies in wheat, but most have used advanced or preliminary breeding lines as a TP (Zhang et al., 2022). However, it was reported that GS successfully used the CC in other crops, such as pepper (Hong et al., 2020). In genomic selection, prediction accuracy is affected by various factors, such as assumptions of prediction methods, markers, and training populations. Therefore, we investigated various genomic prediction methods through 10-fold cross-validation. Although there were differences between traits depending on the predictive model, RF showed consistently good prediction accuracy across all traits. The average culm length prediction accuracy was lower than that of other traits, indicating that the genetic structure of the locus is different from other traits. In particular, awn color-ear color and days to heading-days to maturity, which are highly correlated characteristics, showed similar patterns of prediction accuracy. It is consistent with the results of previous studies showing similar patterns among highly correlated traits (Hong et al., 2020). Reports show that heritability is one factor that significantly influences GS (Desta and Ortiz, 2014). However, our study did not show a correlation between prediction accuracy and heritability. HD (0.81) showed a high prediction accuracy but also had a prediction accuracy of 0.6 or higher for most other traits with low heritability. Therefore, this study did not identify a reliable correlation between prediction accuracy and heritability.

Next, the prediction accuracy according to TPs was investigated. It is known that prediction accuracy increases with a large number of training populations. However, it was reported that the genetic diversity of the training population affects prediction accuracy (Edwards et al., 2019). Therefore, when using as diverse training populations, we evaluated the prediction accuracy of clustered accessions according to the population structure. In all traits, it was confirmed that the prediction accuracy increased as the size increased. However, subgroups based on population structure, irrespective of their number, showed different prediction accuracies for all traits. Also, traits that showed a high positive correlation between phenotypes showed a similar pattern in prediction accuracy according to the training population.

Finally, verification was conducted to determine whether the CC’s GS model applied to the validation population. Validation was performed only for the days to heading trait. As a result of applying the validation population consisting of 35 breeding lines to six models, it was possible to confirm the prediction ability of 0.4 or more in the CC-sub 3 training population. Based on the results from previous studies that a prediction accuracy greater than 0.3 would be sufficient to apply genomic selection (Heffner et al., 2011b), our study showed the potential for genomic selection in wheat breeding. The results can potentially provide new molecular marker information associated with those traits based on allelic differences with opposite phenotypes. The maker information obtained from this study should be validated for other breeding populations.

## Data availability statement

The data presented in the study are deposited in the European Nucleotide Archive repository, accession number PRJEB60428 https://www.ebi.ac.uk/ena/browser/view/PRJEB60428.

## Author contributions

Conceptualization, YK and CK; methodology, YK, KM, JK, and C.K.; software, YK; validation, YK, CC, and CK; formal analysis, YK; investigation, KM, JK, and CC: resources, JK, KM,and CC; data curation, YK; writing—original draft preparation, YK; writing—review and editing, YK and CK; visualization, YK; project administration, CK. All authors contributed to the article and approved the submitted version.
