# Supplementary material for: Optimizing genomic selection of agricultural traits using K-wheat core collection
Source: Front Plant Sci. 2023 Jun 14;14:1112297. doi: 10.3389/fpls.2023.1112297 (PMC10303932; doi:10.3389/fpls.2023.1112297)
Supplement: Supplementary file 1 [file Image_1.pdf]

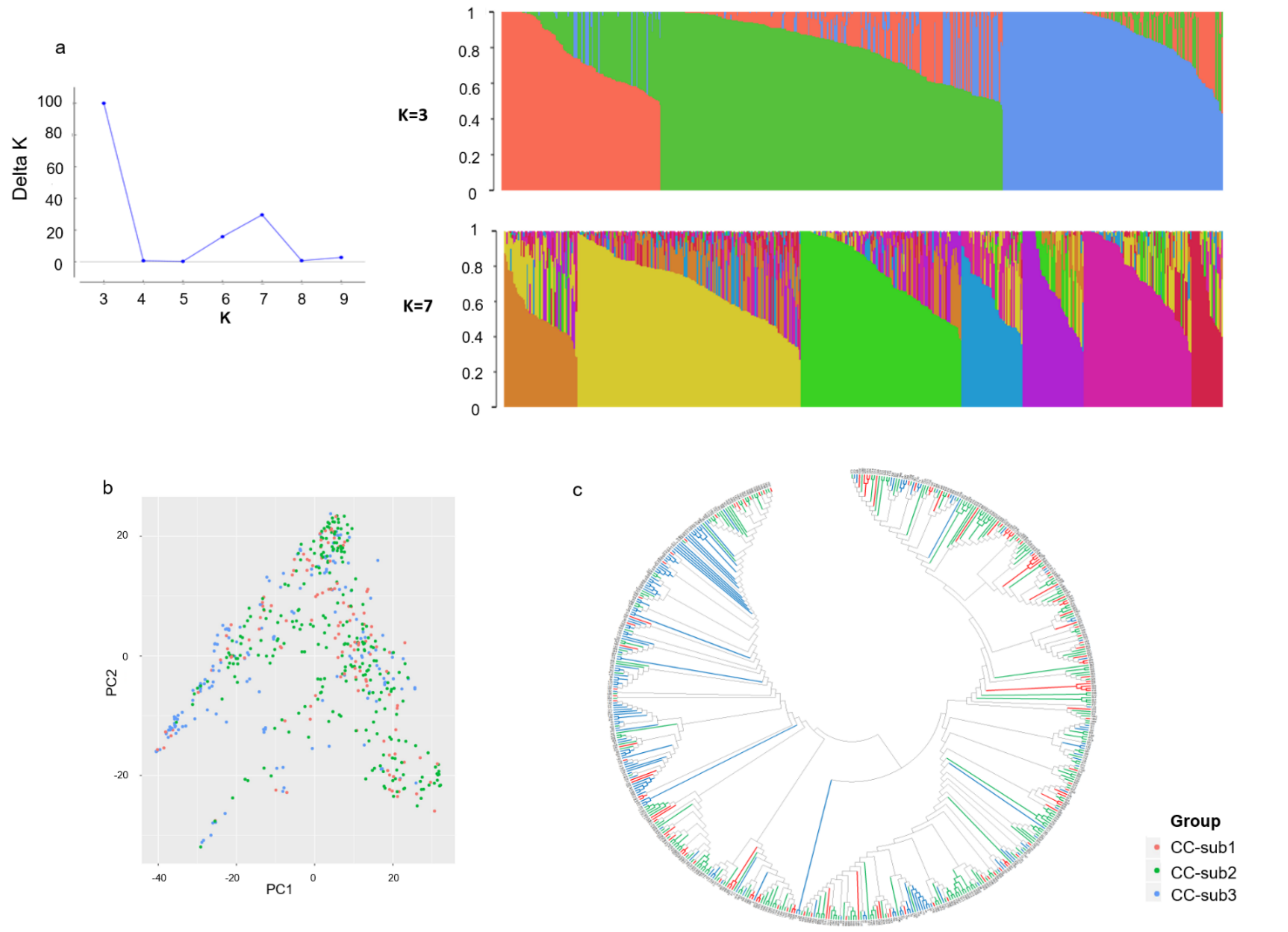

Supplementary Figures 1. Population structure analysis of K-wheat core collection using SNP markers. a) Evanno test and population structure results of the of CC. The population structure shows k=3 and a=7. b) Principal component analysis (PCA) of the CC based on SNP markers. Based on the subpopulation structures (K=3), the red, green, and blue dots represent CC-sub 1, CC-sub2, and CC-sub3, respectively. c) Molecular phylogenetic analysis by Maximum Likelihood method using the core collection. Phylogenetic analysis was inferred by using the Maximum Likelihood method based on the Tamura-Nei model. Phylogeny tree analyses were conducted by MEGA7 (Kumar et al., 2016). Based on the subpopulation structures (K=3), the red, green, and blue lines indicate CC-sub 1, CC-sub2, and CC-sub3, respectively.
