# Supplementary material for: Optimizing genomic selection of agricultural traits using K-wheat core collection
Source: Front Plant Sci. 2023 Jun 14;14:1112297. doi: 10.3389/fpls.2023.1112297 (PMC10303932; doi:10.3389/fpls.2023.1112297)
Supplement: Supplementary file 3 [file Table_2.docx]

Supplementary Table 2. The number of accessions by continent of the K-wheat CC and the K-wheat mini CC.

| Continent | K-wheat CC | K-wheat mini CC |
| --- | --- | --- |
| Africa | 27 (4.76%) | 14 (5.67%) |
| Asia | 229 (40.38%) | 92 (37.25%) |
| Europe | 78 (13.75%) | 43 (17.41%) |
| North America | 61 (10.75%) | 30 (12.15%) |
| South America | 114 (20.10%) | 42 (17%) |
| Oceania | 2 (0.35%) | 4 (1.65%) |
| Unknown | 56 (9.87%) | 22 (8.91%) |
| Total | 567 | 247 |
